# Differences in the Structure of the Gut Bacteria Communities in Development Stages of the Chinese White Pine Beetle (Dendroctonus armandi)

**DOI:** 10.3390/ijms141021006

**Published:** 2013-10-18

**Authors:** Xia Hu, Chunyan Wang, Hui Chen, Junning Ma

**Affiliations:** College of Forestry, Northwest A&F University, Yangling 712100, Shaanxi, China; E-Mails: lake-autumn@163.com (X.H.); chunyan@nwsuaf.edu.cn (C.W.); mjn404@nwsuaf.edu.cn (J.M.)

**Keywords:** bark beetle, bacterial community, symbiosis, DGGE, *Dendroctonus armandi*

## Abstract

The Chinese white pine beetle *Dendroctonus armandi* Tsai and Li, is arguably the most destructive forest insect in the Qinling Mountains in Northern China. Little is known about the structure of the bacterial communities associated with *D. armandi* even though this wood-boring insect plays important roles in ecosystem and biological invasion processes that result in huge economic losses in pine forests. The aim of this study was to investigate the composition of the bacterial communities present in the guts of *D. armandi* at different developmental stages using a culture-independent method involving PCR-denaturing gradient gel electrophoresis (DGGE). Analysis of PCR-amplified 16S rRNA gene fragments of bacteria from the guts of larvae, pupae, and male and female adults revealed bacterial communities of low complexity that differed according to the developmental stage. *Citrobacter* spp. and *Pantoea* spp. predominated in larvae and adults, whereas *Methylobacterium* was the dominant genus at the pupal stage. The main difference between the guts of male and female adults was the greater dominance of *Citrobacter* in females. Previous studies suggest that the bacterial community associated with *D. armandi* guts may influence insect development. The data obtained in this study regarding the phylogenetic relationships and the community structure of intestinal bacteria at different developmental stages of the *D. armandi* life cycle contribute to our understanding of *D. armandi* and could aid the development of new pest control strategies.

## 1. Introduction

Bark beetles, especially *Dendroctonus* species, are considered to be serious pests in coniferous forests [1]. They cause extensive damage to coniferous trees, which results in huge economic losses, and represent a major disturbance factor for the ecosystem [2]. Bark beetles can harbor gut microbial communities that range from simple to complex [3,4]. In a few systems that have been studied in more detail, important contributions to the host insect physiology and life history have been attributed to the gut-associated microbes [5,6]. The relationship between the nutrient-poor substrate on which the wood-boring insects feed and the cellulolytic and nitrogen-fixing microbes that are associated with these insects has also been investigated [7,8].

Chinese white pine beetle (*Dendroctonus armandi* Tsai and Li, Scolytidae), an important pest in the Qinling and Bashan Mountains in Northern China, is able to kill living *Pinus armandi* and has caused serious damage to *P. armandi* forests since 1954 [9]. Owing to the importance of *D. armandi*, various aspects of its biology and physiology have been studied, such as its niche within the *P. armandi* ecosystem [9], its life cycle [10] and symbiotic fungi [11]. However, little is known about its symbiotic intestinal bacterial communities.

Bacterial communities are known to play important roles in the life histories of bark beetles, contributing to their reproductive success, community interactions and niche diversification [12], as well as their development and survival in harsh environments by metabolizing toxins and providing protection against natural enemies [13]. Bacteria can also contribute to the fitness of bark beetles, particularly insects that rely on a nutrient-poor food source, by providing nutritional supplements that are absent from the diet of the insect, such as amino acids [14], essential vitamins [15] and nitrogen and carbon compounds [16,17]. Such relationships seem to be especially prevalent among insects that exploit woody substrates, which are relatively nutrient poor, heavily chemically defended, and available to competitors once the defenses of the woody substrate are depleted [18–20]. Host insects that are deprived of their symbiont show retarded growth, increased mortality and sterility [21]. Some endosymbionts have shown spatial and geographic variation [22,23]; however, it is poorly understood whether the composition of endosymbionts varies among the different developmental stages and between sexes. Hosokawa *et al*. first reported such a case in stinkbugs of the family Plataspidae, wherein specific gut bacteria were vertically transmitted via a “symbiont capsule” [24]. We hypothesized that beetles from all samples in our study would share a small number of bacterial species. We further hypothesized that there would be some relationships between bacterial community structure and the stage of development. The first step toward understanding the roles of endosymbiont community members in bark beetle biology and in the functioning of the community itself is to define the members of the community [25].

In our study, gut-associated bacterial communities of *D. armandi* in the larval, pupal and adult stages were investigated using a culture-independent method. We quantified the variability of bacterial communities and determined the frequency of specific bacterial phylotypes associated with the three developmental stages. Components of the bacterial community profiles were excised and sequenced to determine the identity of the taxa using PCR-denaturing gradient gel electrophoresis (DGGE) [26].

## 2. Results and Discussion

### 2.1. Bacterial Diversity Analysis

**Figure 1:**
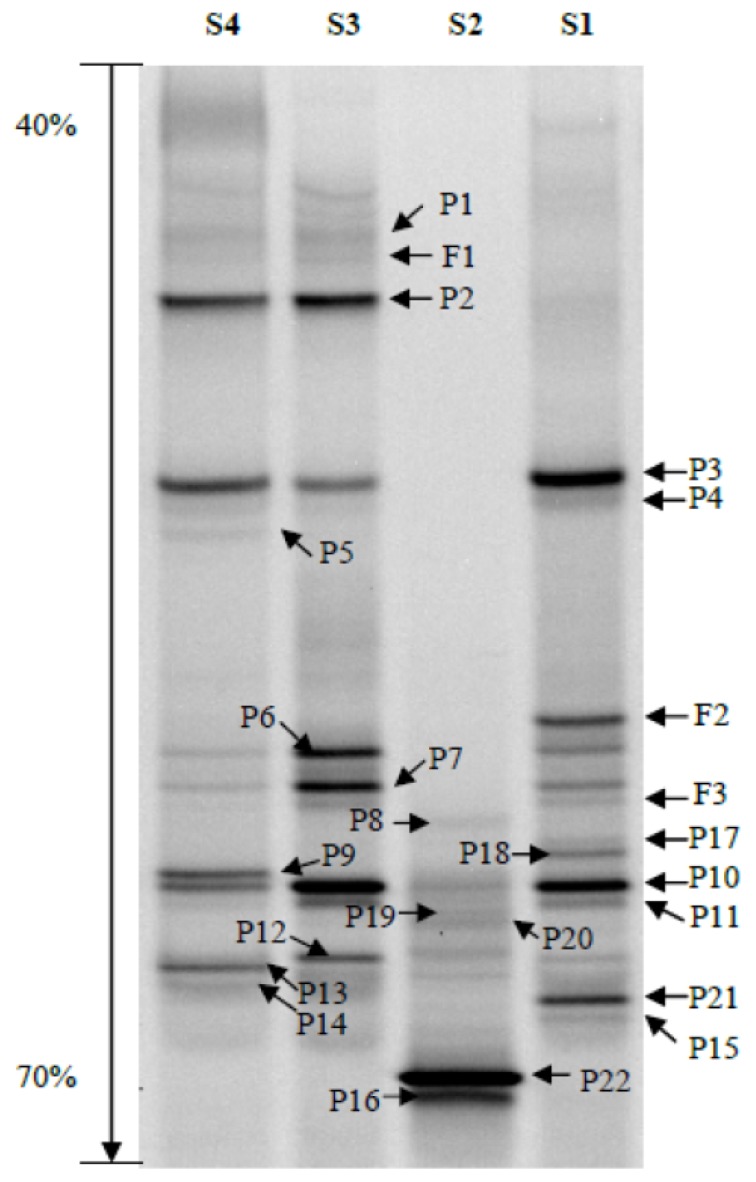
Denaturing gradient gel electrophoresis (DGGE) profiles of nested PCR-amplified 16Sr RNA gene fragments of bacteria from the guts of *Dendroctonus armandi* larvae (**lane S1**), pupae (**S2**), adult females (**S3**), and adult males (**S4**). Bands P1–P22 and F1–F3 represent 16S rRNA gene regions of different bacteria.

The number, density and composition of bacterial DGGE bands were different in each sample (Figure 1). Based on the peak density of bands in the DGGE profile, the bacterial diversity indices were analyzed as a way of estimating the diversity of microbial communities, which showed that the value of the Shannon–Wiener index (*H*′) was positively related to the diversity of the bacterial community. The richness and *H*′ value (2.367476) of bacteria in the guts of *D. armandi* larvae (S1) were the highest with 16 visible bands in the DGGE profile, whereas the pupal stage (S2) was the lowest with only 10 visible bands (Table 1). There was no significant difference between the bacterial diversity of the larval and adult stages (*p* > 0.05), or that of the male and female adults (*p* > 0.05). The *H*′ value of gut bacteria found in the pupal stage was significantly different compared with those of the larval and adult stages (*p* < 0.05).

### 2.2. Phylogenetic Analyses and Dominant Taxa

**Figure 2:**
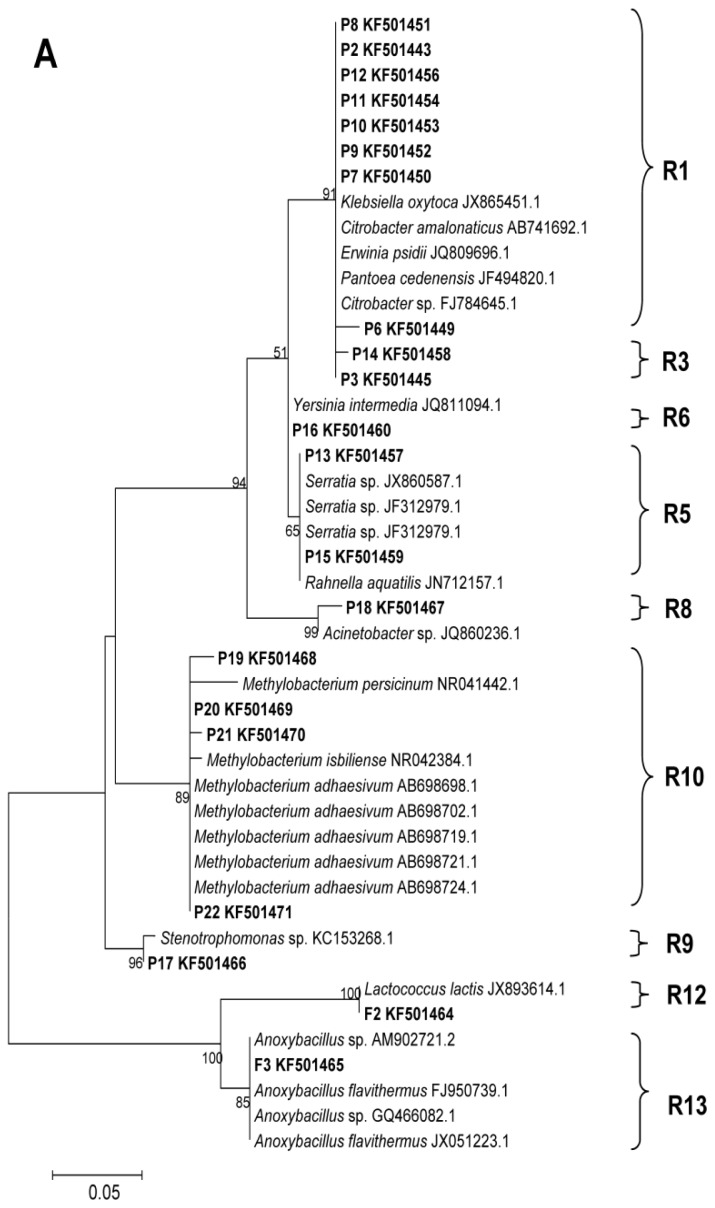

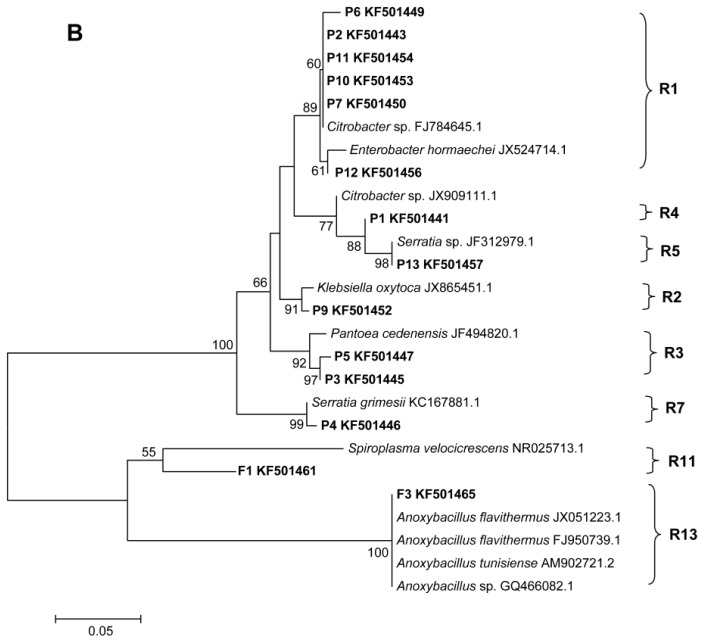
Phylogenetic trees of the bacterial communities in the gut of *Dendroctonus armandi* based on the 16S rRNA gene fragments. (**A**) Maximum likelihood phylogenetic tree of the bacterial community in the guts of larvae and pupae using the model Jukes-Cantor + I model; (**B**) Neighbor-joining phylogenetic tree of the bacterial community in the guts of female and male adults using the Kimura 2-parameter + G model. R1–R13 represent different operational taxonomic units with a 3% sequence dissimilarity cut-off (OTUs_0.03_) cluster obtained with MOTHUR.

Each of the distinguishable bands in the separation pattern represented an individual bacterial species [27]. In total, 25 different bands were successfully sequenced and two phylogenetic trees were constructed for the larvae and pupae (Figure 2A), and male and female adults (Figure 2B). Sequence identification revealed that the majority of species were Proteobacteria (γ-Proteobacteria, labeled P1–P18 and α-Proteobacteria, labeled P19–P22 in Figure 1) and a few species were Firmicutes (labeled F1–F3 in Figure 1).

*Citrobacter* spp. and *Pantoea* spp. (γ-Proteobacteria, Enterobacteriaceae) were the predominant bacteria in the guts of larval *D. armandi*, and *Methylobacterium* (α-Proteobacteria) predominated in the pupal guts. Both female and male adults contained a high proportion of *Citrobacter*. P2, P6, P7 and P10, which all belong to the *Citrobacter* genus, were the most distinct bands in the DGGE profile of bacteria from the guts of adult females (Figure 1). Band P3, which was identified as *Pantoea*, was the second most common bacteria in the guts of male adults (Figure 1).

### 2.3. OTUs

According to distance matrices of the sequences, 13 OTUs_0.03_ clusters were obtained (R1–R13). R1 and R5 OTUs_0.03_ clusters were observed in the gut bacteria of all the samples (Figure 2). Sequences belonging to the R1 and R5 clusters grouped together on the same phylogenetic branch, and were closely related to *Citrobacter* and *Serratia*, respectively. In addition, cluster R1 was the most dominant group in all the samples except those from pupae, which were dominated by the R10 cluster, which comprised *Methylobacterium* spp.

**Figure 3:**
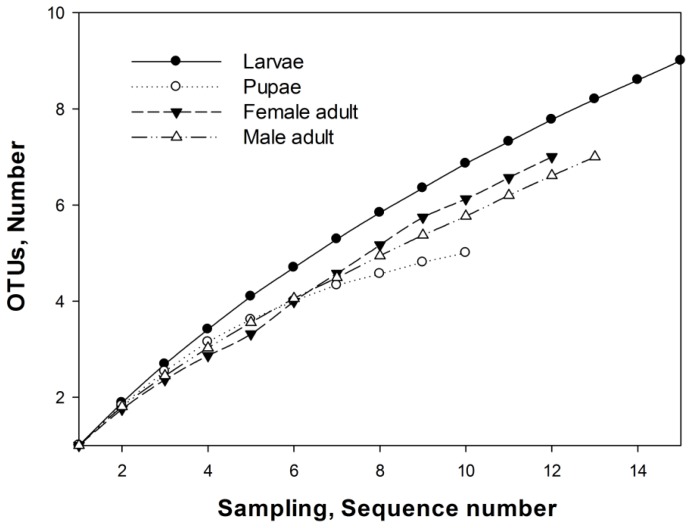
Rarefaction curves of bacterial 16S rRNA sequences in the gut of *Dendroctonus armandi* larvae, pupae, adult females and males, calculated using MOTHUR OUTs_0.03_. The plot showed the number of new bacterial species as a function of the number of clones sequenced.

The Chao index was used to better characterize the relative bacterial species richness of the different developmental stages and sexes of *D. armandi*. The OTU and richness estimation data revealed that the bacterial community structure was simplest in the pupal guts (Figure 3), with five OTUs_0.03_ clusters (R1, R2, R5, R6, R10) belonging to γ-Proteobacteria and α-Proteobacteria (Figure 2A). Bacterial species richness was highest in larval guts, with nine OTUs_0.03_ (R1, R3, R5, R7, R8, R9, R10, R12, R13) belonging to γ-Proteobacteria, α-Proteobacteria and Firmicutes (Figure 2A). Seven OTUs_0.03_ clusters (Figure 2B), all belonging to Enterobacteriaceae (γ-Proteobacteria), were found in the guts of female adults (R1, R3, R4, R5, R7, R10, R13) and male adults (R1, R2, R3, R4, R5, R7, R10).

### 2.4. Bacterial Community Structure

This study has provided new insight into the gut bacterial diversity of *D. armandi* at different developmental stages and in the different sexes. Overall, the results of the bacterial diversity indices were consistent with the OTU richness estimation. The predominant species found in the intestinal bacterial communities of *D. armandi* formed a group of low complexity; however, the structure of the bacterial community differed depending on the developmental stage. A low level of bacterial community complexity in insect guts seems to be the norm [28–32], although there are some exceptions, for example, termite guts [33]. As we hypothesized, the gut-associated bacteria from all samples comprised only a few bacterial species. *Citrobacter* (R1) and *Serratia* (R5), which belong to the Enterobacteriaceae (γ-Proteobacteria), were present in all samples from *D. armandi* guts. These conserved bacterial communities of shared bacterial taxa should be well adapted to their host. Members of the Enterobacteriaceae are commonly found in the gut communities of a wide range of animals, including humans and insects, and can aid in vitamin biosynthesis, pheromone production and degradation of plant compounds [34,35].

*Citrobacter* and *Pantoea* were the most abundant taxa in the guts of larvae and adults. The highest levels of *Citrobacter* were found in the guts of female adults. A range of different bacterial communities have been reported to be associated with bark beetles (*Dendroctonus* spp.), including *Citrobacter* spp. and *Pantoea* spp. [20,36–38]. Previous studies have reported that *Citrobacter* spp. and *Pantoea* spp. are associated with nitrogen fixation in the guts of termites and fruit flies (*Ceratitis capitata*) [39–41]. The abundance of *Citrobacter* in our study suggested that the nitrogen-fixing process could be an important dietary supplement of assimilable nitrogen for the bark beetle. In addition, *Citrobacter* sp. has also been shown to affect insect oviposition and larval development [42,43], which might explain why the guts of adult females contained the highest levels of *Citrobacter*. To clarify the *Citrobacter*-*Dendroctonus* relationship, more studies are necessary to determine the nutritional status of this nitrogen-fixing bacterium in bark beetles.

Among the developmental stages investigated in this study, the pupal stage showed the simplest bacterial structure according to both the diversity indices and the Chao index. These results support previous findings of low bacterial diversity in the pupal guts of bark beetles [44].

Interestingly, *Methylobacterium* was the dominant taxa in the pupal stage. Species in the *Methylobacterium* genus are facultatively methylotrophic bacteria, and are mostly found in soil, as well as in the xylem and in other parts of plants [45,46]. They can use a variety of organic substrates with carbon-carbon bonds as sources of carbon and energy [47]. Most studies of the bacteria in this genus have focused on their roles in methane oxidization and the serine cycle. The role of gut-associated *Methylobacterium* spp. in *D. armandi* might be similar to that reported in studies of leafhopper and weevil, which feed on a complex carbon source [45,46]. Energy is required to achieve metamorphosis through the activation of several metabolic processes and morphological changes even though pupae do not feed. Therefore, the decline of bacterial diversity and the dominance of *Methylobacterium* spp. in pupae in this study could be associated with the absence of feeding activity and the necessity for pupae to extract the remaining energy from undigested food through the action of *Methylobacterium* spp. *Methylobacterium* spp. play an important role in the utilization of organic carbon sources and conversion of amino acids to support physiological activities in the pupal development stage. To clarify the *Methylobacterium*-*Dendroctonus* relationship, further studies are necessary to determine the role of this methane-oxidizing bacterial genus in the bark beetle life cycle.

The bacterial community structures found in the guts of adult males and females were similar, which supports similar findings in an earlier study [47], suggesting that the bacterial community structure in *D. armandi* adults is conserved. The bacterial communities in the adult guts belonged to the γ-Proteobacteria and Bacilli. *Citrobacter* was a dominant genus in both sexes, particularly in female adults. Our data suggest that although some of the bacteria found in the guts of adult bark beetles might relate to their sexual roles, most of the bacteria played similar roles in both sexes.

The information about bacterial community structure collected in this study will provide the basis for subsequent studies on the roles of these intestinal bacteria in bark beetle development, ecology and management. A study of the bacterial flora in the guts of *Dendroctonus rhizophagus* proposed that most of the bacterial genera present could be implicated in nitrogen fixation and cellulose breakdown, which are important roles associated with insect development and fitness, particularly given the challenging environment inhabited by the bark beetles [44].

Gut bacteria have been proposed as a means of achieving pest biocontrol. For example, *Enterobacter gergoviae*, a gut bacterium of the pink bollworm (*Pectinophora gossypiella*), was transformed to express Cyt1A, an insecticidal protein lethal to mosquitoes and black fly larvae [48]. plant surface bacteria have also been considered as biopesticide vectors to deliver insecticidal proteins to phytophagous insects [49]. The possibility of using the gut bacterium *E. cloacae* to control the mulberry pyralid (*Glyphodes pyloalis*) has also been successfully demonstrated by transforming the ice nucleation gene. An increased supercooling point was observed in *G. pyloalis* that had been colonized by the transformed gut bacteria, leading to an increased mortality rate [50]. Transgenic gut bacteria have even been used for the control of insect-borne human diseases, for example, transgenic *Rhodococcus rhodnii* has been used to control the triatomine bug *Rhodnius prolixus*, the vector of the medically important parasite *Trypanosoma cruzi*. [51]. Although there are scarcely any reports about using gut bacteria to control bark beetles, gut bacteria are a potential contender for pest biocontrol strategies in the future. Detailed knowledge about the dynamic variation, colonization and modes of transmission of intestinal bacteria is required before such a strategy could be successfully implemented.

## 3. Experimental Section

### 3.1. Insect Collection and Dissection

Larvae, pupae, and female and male adults of *D. armandi* were collected from the bark of infested *P. armandi* at the Huoditang Experimental Forest Station of the Northwest A&F University in August 2012. The collection site was located on the southern slope of the middle Qinling Mountains (33°18′–33°28′N, 108°21′–108°39′E), Shaanxi, China.

All the *D. armandi* samples were collected manually using fine forceps to extract the insects directly from the galleries of infested pine trees. The samples were transported to the laboratory in sterile vials containing sterile moist paper. To investigate the influence of sex on gut-associated bacteria, female and male adults were separated according to their reproductive organs.

Insect samples were rinsed in sterile water, surface sterilized with 70% ethanol for 3 min, and then rinsed twice in sterile water. After being placed in 10 mM sterilized phosphate-buffered saline (138 mM NaCl and 2.7 mM KCl, pH7.4), the insects were dissected under the stereomicroscope with the aid of insect pins to excise the mid-guts and hindguts [8]. Forty guts were excised from each of the developmental stages. Guts from the same development stage were transferred to a 1.5-mL microcentrifuge tube, ground several times with a plastic pestle in liquid nitrogen, and then vortexed with 500 μL of Tris-EDTA (10 mM Tris-HCl (pH 8.0), 1 mM EDTA) for 3 min at maximum speed (2500 r/min) and then centrifuged at low speed (4000 r/min) for 15 s to separate microbial cells from the gut wall tissues and undigested food. The supernatant (containing bacteria) was transferred to new tubes for DNA extraction. All the procedures were performed in a sterile environment.

### 3.2. Extraction of Bacterial DNA

Bacterial DNA was extracted using the E.Z.N.A. Bacteria DNA Kit (Omega Biotech, Doraville, GA, USA) according to the manufacturer’s directions, and then stored at −20 °C until needed.

### 3.3. Nested PCR

Nested PCR was used to increase the resolution yield of DGGE [52]. The highly variable V3 region of the bacterial 16S rRNA gene was amplified using primer pairs fD1 (5′-AGAGTTTGATCCTGG CTCAG-3′) and rP1 (5′-ACGGTTACCTTGTTACGACTT-3′) in the first round of PCR, and 341F-GC (5′-CGCCCGCCGCGCGCGGCGGGCGGGGCGGGGGCACGGGGGGCCTACGGGAGG CAGCAG-3′) and 534R (5′-ATTACCGCGGCTGCTGG-3′) in the second round of PCR [53]. All PCR amplifications were performed with a S1000™ Thermal Cycler (Bio-Rad, Hercules, CA, USA) in a final mixture volume of 50 μL, containing 25 μL 2× Taq Master Mix (CoWin Biotech, Beijing, China), 0.8 μL of each primer (10 μM, Invitrogen Trading, Beijing, China), 1 μL template and 22 μL RNase-free water.

The bacterial DNA was diluted to 30 ng μL^−1^ and used as the template for the first round of PCR, which was carried out using the following program: 94 °C for 3 min, followed by 30 cycles, each at 94 °C for 1 min, 55 °C for 1 min, 72 °C for 1.5 min and a final extension at 72 °C for 5 min. The product of the first round of PCR was diluted 1/200 with ddH_2_O, and used as the template for the second PCR reaction, which was carried out using the following procedure: 94 °C for 3 min, followed by 30 cycles of 94 °C for 30 s, 56 °C for 30 s, 72 °C for 30 s and a final step at 72 °C for 5 min. The PCR products obtained and the primer specificity were analyzed by 1.2% (*w*/*v*) agarose gel electrophoresis and ethidium bromide staining in the presence of a DL2000 DNA marker (Takara Biotechnology, Dalian, China). The obtained PCR products were stored at −20 °C until DGGE analysis.

### 3.4. Denaturing Gradient Gel Electrophoresis (DGGE)

The DCode™ Universal Mutation Detection System (Bio-Rad) was used for the DGGE analysis. 35 μL of bacterial nested-PCR products from each sample were loaded onto an 8% (*w*/*v*) poly-acrylamide (37.5:1 acrylamide/bio-acrylamide) gel containing a linear denaturing gradient of 40% to 70%, where 100% denaturing acrylamide was defined as containing 7 M urea and 40% formamide [54]. The gel was run at 120 V initially for 10 min, and then 70 V for an additional 11 h at 58 °C in 1× TAE buffer (40 mM Tris-acetate (pH 7.4), 20 mM sodium acetate, 1 mM disodium EDTA). After being stained with ethidium bromide solution for 10 min, the gel was eluted in deionized water for 10 min, and then photographed under UV light by using the Gel Doc™ XR System (Bio-Rad).

### 3.5. DGGE Band Identification

Dominant DGGE bands were excised from the poly-acrylamide gel and the DNA was eluted out using an E.Z.N.A. Mag-Bind Poly-Gel DNA Extraction Kit (Omega). The eluted DNA (1 μL) was re-amplified with primer pair 534R and 341F without a GC clamp at the 5′ end. The PCR products were purified using a Gel Extraction Kit (Baitaike Biological Technology, Xi’an, China), and connected to the plasmid pMD18-T vector (pMD18-T Cloning Kit, Takara Biotechnology, Dalian, China). The recombinant plasmids were transformed into *Escherichia coli* (strain DH5α) and the positive colonies were identified based on the blue-white screening. Five positive colonies were selected randomly from each transformation to further confirm the presence of correct inserts via PCR with the primer pair M13-47 and M13-48. The confirmed clones were sequenced (Jinsirui Biotechnology, Nanjing, China).

In order to determine the classification status of the intestinal bacteria, the sequences were matched with sequences in the RDP II database [55], and blasted in the NCBI database [56] and the EzTaxon-e database [57] to select and download the sequences that were reliable and that had a high sequence similarity. All the sequences obtained in this study have been submitted to the NCBI database (accession numbers KF501441–KF501471). Phylogenetic relationships among the intestinal bacteria were analyzed using molecular phylogeny techniques. Sequences were aligned using MUSCLE [58], available in the software MEGA 5, which computed the best model. The phylogenetic trees were constructed using Maximum Likelihood and Neighbor-joining methods [59]. To calculate the support for each clade, bootstrap analysis was performed with 1000 replications.

### 3.6. DGGE Band Profile Analysis

The following procedure was used to analyze the DGGE band profiles using Quantity One software (Bio-Rad). First the auto frame lanes were selected and the rolling disk size was adjusted to five to minimize the influence of background. Second, the bands were detected and the parameters adjusted to acquire the most reliable band pattern, and then the Gauss-model was applied to all the lanes. Third, the lane with the most bands was selected using auto-match and the tolerance was set at 4.00%; the other lanes were matched manually. Fourth, the peak density of all the lanes was reported for further analysis.

Each band was digitized via auto detection of peak density. Based on the transferred data, the diversity indices were calculated to investigate the dominant bacterial communities and to determine how they changed in the larvae, pupae, and adult females and males. Various indices of biodiversity, such as the Shannon–Wiener index (*H*′), Richness (*S*) and Evenness (*E_H_*), were calculated from the DGGE patterns according to the following equations:

(1) $$H^{\prime }=-\sum_{i=1}^{S}p_{i}\;\text{ln }p_{i}=-\sum_{i=1}^{S}\left(N_{i}/N)\;\text{ln}(N_{i}/N\right)$$

(2) $$E_{H}=H/H_{\text{max}}=H/\text{ln }S$$

where *S* is the number of bands in a lane, *Ni* is the peak density of the *i*th band and *N* is the total peak density of all bands in a lane [60,61]. The significant differences between means were analyzed by *t*-test in SPSS Version 18.0 (SPSS Inc., Chicago, IL, USA).

### 3.7. Operational Taxonomic Units and Richness Estimation

Sequences in each phylogenetic tree were formatted to FASTA files and used to construct distance matrices for each library with MOTHUR Version 1.29.0 (Patrick Schloss, The University of Michigan, Ann Arbor, MI, USA). The distance matrices were used as the input files to define the OTUs on the basis of a similarity distance cutoff of 0.03. OTUs defined by distances of 0.03 were generally corresponded to a species [62]. Sequences belonging to the same cluster based on the reference of OTUs_0.03_ were grouped together in the phylogenic trees and labeled R1–R13 for the purpose of clarity (Figure 2). The Chao index [63] was calculated to measure the absolute value of species richness. Rarefaction curve methodology was used [64,65] to estimate the relationship between the expected OTU richness and sampling depth. Finally, the rarefaction curves were generated using SigmaPlot Version 10.1 (Systat Software, Inc., San Jose, CA, USA).

## 4. Conclusions

This study revealed the structure of the gut-associated bacterial communities in the different developmental stages of *D. armandi* collected from the southern slope of the middle Qinling Mountains in August 2012. The predominant bacterial species varied during the life cycle of *D. armandi*. Several important bacteria were identified, including nitrogen-fixing and carbon transformation bacteria (*Citrobacter*, *Pantoea* and *Methylobacterium*), which are likely to play important roles at different developmental stages of the bark beetle. We propose that gut-associated bacteria could interfere with the development of *D. armandi* and, hence, may have potential as a vector for a biocontrol agent.

## Figures and Tables

**Table 1:** Richness (*S*), Evenness (*E_H_*) and Shannon-Wiener index (*H*′) of intestinal symbiotic bacteria identified in samples of *Dendroctonus armandi* at different developmental stages.

Lane	*D. armandi* sample	*S*	*E_H_*	*H*′
S1	Larvae	16	0.853886	2.367476
S2	Pupae	10	0.649392	1.49528
S3	Female adult	12	0.854255	2.122745
S4	Male adult	14	0.885312	2.336389
